# A Fusion of Entropy-Enhanced Image Processing and Improved YOLOv8 for Smoke Recognition in Mine Fires

**DOI:** 10.3390/e27080791

**Published:** 2025-07-25

**Authors:** Xiaowei Li, Yi Liu

**Affiliations:** School of Mechanical Electronic and Information Engineering, China University of Mining and Technology (Beijing), Beijing 100083, China; liuyi@cumtb.edu.cn

**Keywords:** fire smoke, entropy-enhanced images, isometric frame image differencing, entropy-constrained features, smoke recognition

## Abstract

Smoke appears earlier than flames, so image-based fire monitoring techniques mainly focus on the detection of smoke, which is regarded as one of the effective strategies for preventing the spread of initial fires that eventually evolve into serious fires. Smoke monitoring in mine fires faces serious challenges: the underground environment is complex, with smoke and backgrounds being highly integrated and visual features being blurred, which makes it difficult for existing image-based monitoring techniques to meet the actual needs in terms of accuracy and robustness. The conventional ground-based methods are directly used in the underground with a high rate of missed detection and false detection. Aiming at the core problems of mixed target and background information and high boundary uncertainty in smoke images, this paper, inspired by the principle of information entropy, proposes a method for recognizing smoke from mine fires by integrating entropy-enhanced image processing and improved YOLOv8. Firstly, according to the entropy change characteristics of spatio-temporal information brought by smoke diffusion movement, based on spatio-temporal entropy separation, an equidistant frame image differential fusion method is proposed, which effectively suppresses the low entropy background noise, enhances the detail clarity of the high entropy smoke region, and significantly improves the image signal-to-noise ratio. Further, in order to cope with the variable scale and complex texture (high information entropy) of the smoke target, an improvement mechanism based on entropy-constrained feature focusing is introduced on the basis of the YOLOv8m model, so as to more effectively capture and distinguish the rich detailed features and uncertain information of the smoke region, realizing the balanced and accurate detection of large and small smoke targets. The experiments show that the comprehensive performance of the proposed method is significantly better than the baseline model and similar algorithms, and it can meet the demand of real-time detection. Compared with YOLOv9m, YOLOv10n, and YOLOv11n, although there is a decrease in inference speed, the accuracy, recall, average detection accuracy *mAP* (50), and *mAP* (50–95) performance metrics are all substantially improved. The precision and robustness of smoke recognition in complex mine scenarios are effectively improved.

## 1. Introduction

Coal has accounted for the highest proportion of the past energy consumption structure in China, and safety production is the most important issue facing coal mining [1,2,3,4]. The incidence of coal mine accidents is still high despite widespread attention [5,6,7,8]. Fire is one of the accidents that seriously affect the safe production of coal mines [9,10]. Fire accidents have caused a large number of casualties and equipment damage [11,12,13]. On 24 September 2023, a transportation tape friction fire accident occurred in the underground of Panjiang Fine Coal Co., Ltd.’s Shanjiaoshu Coal Mine in Guizhou Province, resulting in 16 deaths, three injuries, and CNY 42,338,200 of direct economic losses [14]; on 9 May 2023, a large fire accident occurred in the Gengcun coal mine of Henan Sanmenxia Henan Dayou Energy Co., Ltd., resulting in five deaths and a direct economic loss of CNY 14,832,600 [15]; on 4 December 2020, a major fire accident occurred in the Diaoshuidong coal mine in Yongchuan District, Chongqing, resulting in 23 deaths, one serious injury, and a direct economic loss of CNY 26,320,000 [16]; on 27 September 2020, a major fire accident occurred in Songzao coal mine of Chongqing Nengtou Yuxin Energy Co., Ltd., resulting in 16 deaths, 42 injuries, and a direct economic loss of CNY 25.01 million [17]; Accident data studies have shown that less than 20% of the deaths of people are due to burns and trauma after mine fire accidents, while asphyxiation deaths due to insufficient combustion products (CO and CO_2_) account for up to 80% or more [18].

Coal mining enterprises attach great importance to fire prevention and control and have adopted a large number of mine fire monitoring strategies, such as sensor-based monitoring, distributed fiber-optic monitoring, and image monitoring [18,19]. Among them, sensor-based monitoring includes temperature, smoke, and gas monitoring. However, all such detection methods need to be installed near the intended area and have the disadvantages of a small monitoring range in a single unit and a serious lag in response time, which is affected by air transportation time. Distributed fiber-optic temperature monitoring is mostly used for a wide range of multi-point temperature measurement placements, such as underground conveyor belts, cables, and electrical equipment temperature monitoring, but the fiber-optic cable is easily damaged, with installation and maintenance difficulties. Image fire monitoring technology can be divided into flame monitoring and smoke monitoring according to the monitoring object. This type of method belongs to face monitoring, has the advantages of a wide monitoring range, fast response speed, and can be monitored from a long distance; therefore, it is applied to mine fire monitoring by a large number of researchers.

Smoke appears earlier than flames; therefore, image-based fire monitoring techniques focusing on monitoring smoke represent one of the effective means to prevent the further expansion of the initial open flames that can later lead to fires [20,21]. In the early fire smoke monitoring technology based on visual image processing, researchers based on manually extracting static and dynamic features such as color, texture, and the shape of smoke, and then fed these features into feature classifiers (e.g., SVM) for fire smoke recognition [22,23,24,25]. This type of method in the experimental environment can achieve a better detection effect, but the effect is dependent on the accurate manual extraction of smoke features. However, such detection methods have some limitations when facing very complex and changing fire environments. For example, these methods are sensitive to the environmental conditions, and if the difference between the coal mine underground fire smoke itself and the background environment is not obvious, the extraction of smoke detail features is particularly difficult, and recognition is prone to cause omission and misdetection.

With the development of deep learning techniques, a series of deep learning models, such as Convolutional Neural Network (CNN), Faster R-CNN, YOLOv5, and other models, have been used for automatic feature extraction and subsequent fire smoke detection [26,27,28]. Hu et al. [29] proposed a multidirectional smoke detection method based on a value-switching attention mechanism and hybrid NMS, which combines multidirectional detection, soft-pool spatial pyramid pooling, and a weighting strategy to improve the accuracy of early smoke recognition in forest fires. Yuan et al. [30] proposed a novel deep multiscale CNN (DMCNN) for smoke recognition. It consists of several parallel convolutional layers with different kernel sizes to completely extract scale-invariant features. Yin et al. [31] proposed an improved lightweight network combining an attention mechanism and an improved upsampling algorithm to detect smoke based on the YOLOv5s model. The experimental results in the literature show that the detection model shows better results. Although image-based fire and smoke monitoring techniques have been widely studied, the accuracy and robustness of the existing techniques face difficulties in meeting the demand for popularization and application in real and complex mine fire and smoke scenarios.

Current smoke monitoring methods are mostly confined to ground scenes in steady-state environments, and their core defects lie in the information structure level: mine fire smoke forms a high-entropy fusion state with the background in special mining environments, which leads to the blurring of the information boundary between the target and the background, i.e., entropy value convergence. If the conventional monitoring method is directly used, the dynamic high-entropy features of smoke diffusion will be swallowed by the static background, resulting in the absence of key information in the image and causing missed detection; the low entropy background texture is misjudged as the critical region of smoke, resulting in the generalization of noise information and causing false detection. To address the above problems, this paper is inspired by the principle of information entropy; first, according to the entropy change of spatio-temporal information brought by smoke diffusion movement (based on the separation of spatio-temporal entropy), it is proposed to capture the entropy change gradient of smoke diffusion in the temporal dimension through the equidistant frame difference image fusion method so as to realize the structural decoupling of the inhibition of background low-entropy region and the enhancement of the high-entropy region of smoke. This method makes the static background noise effectively suppressed and, at the same time, enhances the detail clarity of the smoke target image, obtaining the smoke target image with low background noise and clear edges. Furthermore, in order to cope with the characteristics of the smoke targets with variable scales and complex textures (high information entropy), we propose the following method: an improved mechanism based on entropy-constrained feature focusing (ECFF) and the YOLOv8m model, which can more effectively capture and distinguish the rich details and uncertainties of the smoke region and realize the balanced and accurate detection of large and small smoke targets. The proposed mechanism is a new approach to the detection of small and large smoke targets. Therefore, we propose a method for mine fire smoke recognition by fusing entropy-enhanced image processing with an improved YOLOv8 model. The method ensures real-time and rapid accurate identification of mine fire smoke and enables rapid disaster warnings to avoid casualties and property damage.

## 2. Algorithm Principles

**Figure 1:**
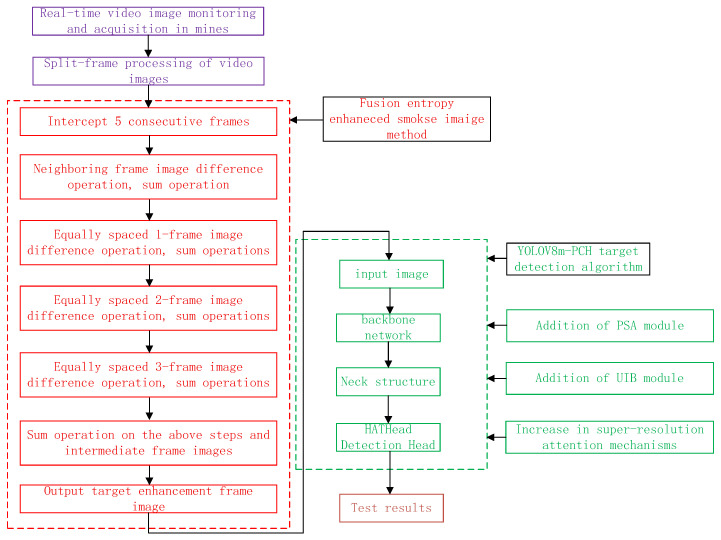
Flowchart of algorithm implementation.

The mine fire smoke recognition algorithm based on fused entropy-enhanced image processing with improved YOLOv8 includes four parts: underground monitoring video image acquisition and processing, isometric frame image difference operation, fused entropy-enhanced smoke imaging, and smoke target frame image recognition. The algorithm implementation process is shown in Figure 1, and the specific implementation steps are as follows:

(1) Capture smoke video images for mine monitoring and process the video images using frames.

Use mine surveillance cameras to capture smoke video in real environments to ensure that the video covers different scenes (e.g., roadways, working faces, equipment areas, etc.). Use video processing tools to extract the video into frame images at a fixed frame rate. Ensure that the framed images are clear and avoid information loss or redundancy due to high or low frame rates.

(2) Perform fusion entropy enhancement on smoke frame images to realize the structural decoupling of (background) low-entropy region suppression and (smoke) high-entropy region enhancement, and construct a smoke image dataset.

According to the entropy change characteristics of spatio-temporal information brought by smoke diffusion movement, based on spatio-temporal entropy separation, the isometric frame image differential fusion method is proposed for smoke image entropy-enhancement processing. The enhanced smoke frame image is labeled, and the location and bounding box of the smoke target are labeled using the LabelImg tool to construct the smoke image dataset. The dataset is divided into training, validation, and test sets (e.g., 6:2:2 ratio) to ensure the scientific nature of model training and evaluation.

(3) Recognizing smoke target image feature information based on the improved YOLOv8 network model.

An improved mechanism based on entropy-constrained feature focusing (ECFF) is introduced into the YOLOv8m model, which can more effectively capture and distinguish the rich details and uncertainties of the smoke region and realize the balanced and accurate detection of large and small smoke targets. Attention mechanisms (e.g., HAT module) are introduced into the YOLOv8m network architecture to enhance the model’s ability to capture smoke features. Train the improved YOLOv8m model using the constructed smoke image dataset. Set the appropriate learning rate, batch size, and number of training rounds to avoid overfitting or underfitting.

(4) Recognizing smoke target frame images.

The trained YOLOv8m improved model is applied to the smoke image to detect the position and bounding box of the smoke target. A suitable confidence threshold is set to balance the detection accuracy and false alarm rate.

## 3. Fire Smoke Fusion Entropy-Enhanced Image Methods

### 3.1. Characterization of Mine Fire Smoke Images

The underground mining environment is special; no natural light source (daylight/moonlight/starlight) leads to the loss of spectral entropy diversity of the environmental light field, and the mine relies entirely on artificial light sources to form a low-entropy lighting base state. The background environment is not as bright as above the mine. It was found that the degradation of underground lighting intensity triggers the degradation of the visual signal-to-noise ratio, and the background texture shows high redundancy, where the smoke forms a strong coupling state, making it difficult to differentiate. Secondly, the smoke from underground fires not only spreads and moves continuously with the development of the fire, but also has the characteristics of stable isotropic diffusion due to the stable ventilation system of the underground and the direction of smoke dispersion in the absence of interference from fire–wind pressure.

**Figure 2:**
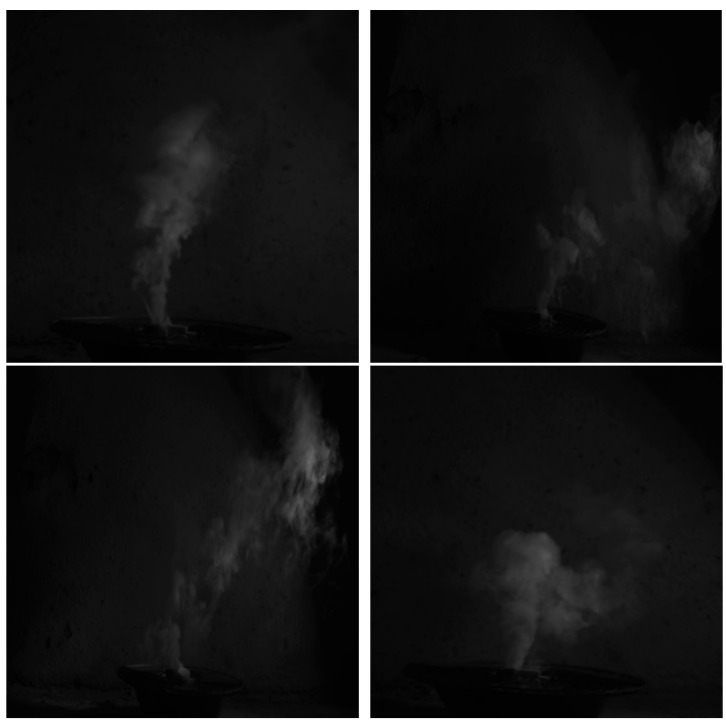
Smoke frame image.

We performed fire smoke video image acquisition in an environment with no natural light source, which was further processed by frame splitting to obtain fire smoke images, as shown in Figure 2.

### 3.2. Principles of Fire Smoke Fusion Entropy-Enhanced Image Methods

It is found that there are brighter lighting environments, such as the main transportation aisle or coal mining face under the mine, but there is a defect of insufficient light in the lighting environments of the access areas; therefore, when the fire smoke appears in the insufficiently illuminated area, the smoke forms a high entropy fusion state with the background in the special mining environment, which results in the blurring of the information boundary between the target and background; it is difficult to differentiate it, so in order to ensure that the high entropy area of the smoke image has a more obvious differentiation from its background (a low-entropy region that has a more obvious distinction), it is necessary to carry out the suppression of the low-entropy region of the background environment and enhance the high-entropy region of the smoke target image so that the smoke target and its boundaries are more obvious in its background environment.

In this paper, we have deeply investigated smoke imagery; the smoke image has dynamic, momentary change, and high-entropy value characteristics, expressed in terms of image features, i.e., the value of the same pixel point in the neighboring frames of the smoke image is large and will change momentarily. On the other hand, the same pixel point values in neighboring frames of underground static background disturbances (e.g., mine walls, coal walls, fixed equipment, etc.) do not have or undergo small changes and have low-entropy value characteristics. Therefore, according to the entropy change characteristics of spatio-temporal information brought by smoke diffusion movement and the entropy invariant or low-change characteristics of background interference information, based on the separation of spatio-temporal entropy, this paper proposes the method of entropy enhancement for smoke target images using equidistant frame images difference fusion. This means periodically intercepting consecutively neighboring M-frames of smoke images from video of smoke, and then, after making neighbor-frames-difference and equidistant-frames-difference operations for M-frames of smoke images, respectively, the obtained neighbor frame image difference and equal interval frame image difference results are summed. The sum result is quadratically summed with the middle frame image of the selected smoke frame image, and finally, the results of the suppression of the background low-entropy region and enhancement of the high-entropy region of the smoke target image are obtained; this makes the static background noise effectively suppressed and, at the same time, enhances the detail clarity of the smoke target image, obtaining a low background noise and clear-edge smoke image. This method effectively suppresses the static background noise, enhances the detail clarity of the smoke target image, and obtains the smoke target image with low background noise and clear edges.

### 3.3. Fusion Entropy-Enhanced Image Method—Frame M-Value Calculation and Algorithm Steps

When the *M*-frame images are even, in the calculation step of the authors’ proposed method of entropy enhancement of the smoke image via the differential fusion of equidistant frame images, two intermediate frame images need to be selected, i.e., $\frac{M}{2}-1$ and $\frac{M}{2}+1$. We sum the two intermediate frame images with the neighboring frame image difference result and the equal interval frame image difference result, and then the target smoke frame image obtained is effectively enhanced, but the background noise is also enhanced at least two times accordingly; therefore, this is not desirable when the M-frame image is even.

From the analysis, it can be seen that, in this paper, the continuous frame image with *M* as an odd number is selected for isometric frame image differential fusion calculation. It is found that, based on the dynamic and high-entropy value characteristics of the mine fire smoke image, the target smoke in the single-frame image has local discretization, a low-entropy value, and is difficult to distinguish when it is similar to the environmental background. When *M* ≤ 3, the smoke target in the obtained frame image will appear as more hollow phenomenon, and the far end of the discrete smoke block and the environmental background is still difficult to distinguish, where the entropy-enhancement effect is not obvious; when *M* ≥ 9, the target smoke in the frame image is enhanced, but the background noise is also enhanced accordingly, and the static interference rejection is not ideal. Therefore, the value of *M*, the number of differential fusion frames of isometric frame images, is selected according to the following formula.

(1) $$M_{1}<M<M_{2},$$

where *M_1_* is the isometric frame image differential fusion frame number minimum, and *M_1_* ≤ 3; *M_2_* is the isometric frame image differential fusion frame number maximum, and *M_2_* ≥ 9.

The number of frames selected is *M* = 5 frames or *M* = 7 frames, and it is found that when the two frame numbers are selected for the entropy enhancement calculation of the smoke target image, whether it is the main body of the smoke target or the discrete smoke block at the far end, compared with its background environment, the smoke target is enhanced in the high-entropy area, and the background noise is suppressed in the low-entropy area; here, the enhancement effect is obvious, but there is not much different between the two, in which the number of frames *M* = 5 is computed more than *M* = 7, and the time consumed is shorter than *M* = 7 (fewer and less time-consuming). Therefore, in this paper, *M* = 5 is selected for the differential fusion entropy enhancement calculation of equidistant frame images.

Five consecutive frames of intercepted smoke images are set as *A_k−_*_2_, *A_k−_*_1_, *A_k_*, *A_k+_*_1_, and *A_k+2_* for calculation. Then, the steps of the entropy-enhancement algorithm for the differential fusion of smoke images with equidistant frame images are as follows:

(1) Perform the pixel difference operation of the neighboring images for each of the five consecutive frames of the intercepted smoke image, and perform the sum operation on the results of the image difference; the calculation formula is shown in (2).

(2) $$P_{1}(m,n)=\sum_{\begin{array}{l} k=3\\ i=j=0 \end{array}}^{\begin{array}{l} k=6\\ i=m\\ j=n \end{array}}\left[{\left(A_{k-1}\right)}_{ij}-{\left(A_{k-2}\right)}_{ij}\right],$$

where *P*_1_(*m, n*) is the result of the sum operation after the differential operation of neighboring frame images; m and *n* are image dimensions; k is the number of image frames; (*A_k−_*_1_)*_ij_* and (*A_k−_*_2_)*_ij_* are the frame image pixel values; *I* and *j* are the row and column coordinates of the image pixel points.

(2) Perform the equal interval 1-frame image pixel difference operation and sum the image difference result; the calculation formula is shown in (3).

(3) $$P_{2}(m,n)=\sum_{\begin{array}{l} k=3\\ i=j=0 \end{array}}^{\begin{array}{l} k=5\\ i=m\\ j=n \end{array}}\left[{\left(A_{k}\right)}_{ij}-{\left(A_{k-2}\right)}_{ij}\right],$$

where *P*_2_(*m, n*) is the result of the sum operation after the differential operation on 1 frame of an image with an equal interval; (*A_k_*)*_ij_* is the image pixel value.

(3) Perform an equally spaced, 2-frame image pixel differencing operation and sum the image differencing results using the formula shown in (4):

(4) $$P_{3}(m,n)=\sum_{\begin{array}{l} k=3\\ i=j=0 \end{array}}^{\begin{array}{l} k=4\\ i=m\\ j=n \end{array}}\left[{\left(A_{k+1}\right)}_{ij}-{\left(A_{k-2}\right)}_{ij}\right],$$

where *P*_3_(*m, n*) is the result of the sum operation after the differential operation of 2 frames of an image with equal intervals; (*A_k+_*_1_)*_ij_* is the pixel value of the frame image.

(4) Perform the pixel difference operation for 3 frames of images with equal intervals and sum the results of the image difference; the calculation formula is shown in (5):

(5) $$P_{4}(m,n)={\left[{\left(A_{k+2}\right)}_{ij}-{\left(A_{k-2}\right)}_{ij}\right]}_{k=3},$$

where *P*_4_(*m, n*) is the result of the sum operation after a differential operation of 3 frames of an image with equal intervals; (*A_k+_*_2_)*_ij_* is the image pixel value.

(5) The neighboring frame image operation results of step (1) are summed with the interval operation results of steps (2), (3), and (4), and the secondary sum operation is performed with the intermediate frame image; then, the image with low-entropy region suppression of the background noise and high-entropy region enhancement of the smoke target is obtained via restoration; the calculation formula is shown in (6):

(6) $$P(m,n)=\sum_{f=1}^{f=4}P_{f(mn)}+{\left(A_{k}\right)}_{ij},$$

where *P*(*m, n*) is the result of the sum operation after the difference operation of the equally spaced frame images and the sum operation of the intermediate frame images, i.e., the fused entropy-enhanced smoke target image; (*A_k_*)*_ij_* is the intermediate frame image pixel value.

### 3.4. Analysis of Fusion Entropy-Enhanced Image Effect and Comparison Results with Existing Methods

**Figure 3:**
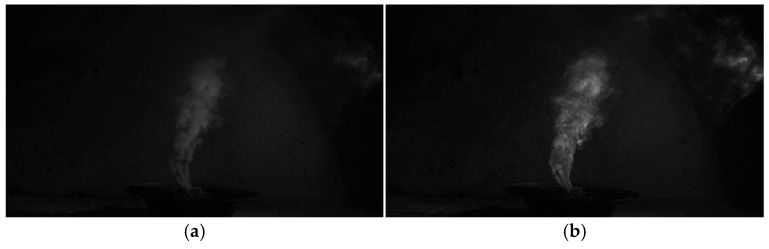
Comparison of smoke image entropy enhancement before and after. (**a**) Original frame image of smoke target; (**b**) fused entropy-enhanced smoke target frame image.

In order to verify the feasibility of this algorithm, the author intercepted five consecutive frames of the captured smoke video images for smoke target image fusion entropy enhancement. The smoke frame image before enhancement, as shown in Figure 3a, is based on the isometric frame image differential fusion smoke image entropy-enhancement method proposed in this paper; the smoke image for the target high-entropy region enhancement, after the enhancement of the smoke target image, is shown in Figure 3b. After the fusion of the entropy-enhanced image before and after the results of the comparative analysis, compared with the original frame image of the smoke target, the entropy-enhanced smoke target contour is clear, and there is a clear distinction between its background environment; secondly, the discrete smoke blocks at the distal end are also well revealed. The method effectively suppresses the low-entropy region of the background noise and enhances the high-entropy region of the target smoke.

**Figure 4:**
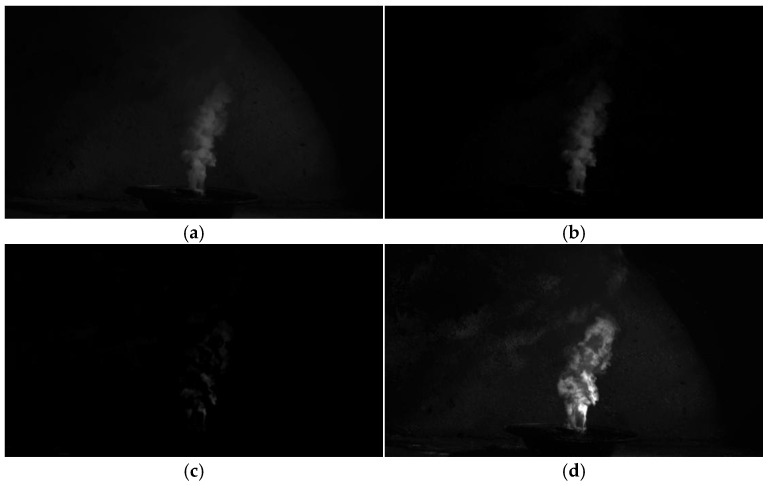
Comparison of entropy-enhancement effect on smoke image. (**a**) Original image of smoke target; (**b**) background subtraction smoke target image; (**c**) smoke target image via frame difference method; (**d**) smoke target image for this method.

Methods to enhance the entropy of the smoke target image by suppressing the interference of the low-entropy region of the background noise include the background subtraction method and the frame difference method [32]. The background subtraction method, i.e., the background difference method, further obtains a noise-reduced smoke target image through the difference operation between the smoke target frame image and the background frame image where the target is located; however, this method is used when there is insufficient background data, which will lead to a large error in the result of the difference operation, and thus, it cannot obtain an accurate smoke target image. The frame difference method uses the continuous video frames for a neighboring frame image difference operation to obtain the target image area. However, the frame difference method has a small or even equal difference in pixel values between neighboring frames, which results in the target image being distributed into multiple discrete regions after the difference operation, which is not conducive to target image recognition. A comparison of the smoke target image-enhancement results obtained based on this method with the background subtraction method and the frame difference method is shown in Figure 4.

As shown in Figure 4, Figure 4b shows the smoke target image obtained using the background subtraction method. Although the smoke image can be clearly seen, the smoke contour and the background are still fused, and the target clarity is only a little better than the original image. Figure 4c shows the smoke target image obtained using the frame difference method. Although the interference of the low-entropy region of the background environment is reduced, at the same time, the detailed features of the smoke are also reduced, and the smoke target image is split into several discrete regions. Figure 4d shows the smoke target image obtained using this method, and it can be seen that the clarity of the smoke image is obviously enhanced under the effective background environment low-entropy region suppression, and even the discrete dispersed smoke region is enhanced. It is clearly superior to the background subtraction method and the frame difference method.

In order to evaluate the enhancement effects (i.e., the enhancement of target legibility and improvement of visual perception quality) more scientifically and in line with the task objectives, we designed and implemented a rigorous subjective user study (User Study). Specifically, we invited 15 experts with experience in computerized image processing and a background in mining. They were presented with the original image, an image enhanced using background subtraction, an image enhanced using frame difference, and an image enhanced using the entropy process of our method. The participants were also asked to judge the images based on three explicit evaluation criteria: target (smoke) clarity, degree of background noise suppression, and overall visual comfort, where the image quality was ranked on a scale (1–5 point scale). Finally, the results were analyzed by using mean opinion score (MOS), where a larger MOS represents a higher quality of the target image. The mean opinion score (MOS) is calculated as shown in the following equation:

(7) $$MOS=\frac{T_{gs}}{N_{gs}},$$

where *T_gs_* is the total score of judging; *Ngs* is the number of judging indicators; and *gs* is the abbreviation of judging indicators.

The results are shown in Table 1, where our method significantly outperforms the comparison method on the core dimension of perceived image quality (*MOS* score: [69.3] vs. [40.0] vs. [7.0]), which strongly supports the intuitive visual advantage demonstrated in Figure 4d.

From the above enhancement algorithm comparison experiments, it can be seen that the isometric frame image differential fusion smoke image entropy-enhancement method proposed in this paper effectively enhances the visibility of the smoke target image without increasing the entropy value of the background environment, and it reduces the interference of the static background (e.g., mine walls, equipment, etc.) with the detection of smoke. The isometric frame image differential fusion strategy proposed in this paper realizes the entropy-enhancement of the smoke target image to improve the accuracy of the downstream detection task, which makes the details of the smoke target image enlarged, the voids of the smoke target image filled, and the discrete blocks of smoke appear more clearly. As a result, the downstream detection module is able to capture the complete smoke contour feature information and is able to recognize it more accurately. However, this method has a limited ability to deal with dynamic interference (e.g., movement of the miners, operation of the equipment, etc.); therefore, the YOLOv8m algorithm was further optimized for the mine fire smoke detection task, and the accuracy and robustness of smoke recognition were further improved through this.

## 4. Improved YOLOv8m-Based Smoke Image Recognition Algorithm

The YOLOv8m algorithm consists of an input, a backbone network, a neck network, and a detection header. The backbone network (backbone) uses the improved CSPDarkNet-53 network; the neck network (neck) introduces the SPPF (Spatial Pyramid Pooling-Fast) [33] module, and the feature fusion module is FPN-PAN (Feature Pyramid Network-Path Aggregation network) [34,35]; the detection head (head) part uses a decoupled detection head to split the target classification and bounding box regression tasks into two independent branches, which improves algorithm performance and convergence speed.

### 4.1. Algorithm Introduction and Improvement Mechanisms

Being able to accurately and quickly identify mine fire smoke is an important measure to avoid or respond to disasters in time, where mine fire smoke detection is typically a time-sensitive task; however, existing algorithms generally adopt complex algorithmic structures due to the pursuit of detection accuracy, which leads to reduced inference speed, thus seriously affecting the real-time nature of smoke detection.

YOLOv8m can be efficiently used for real-time object detection as a one-stage target detection algorithm that predicts the class and location of an object directly from the input image without an additional region proposal step. The attention mechanism can further improve model inference performance and give it significant global modeling capability. Inspired by the principle of maximum information entropy [36,37], this paper proposes an entropy-constrained feature focusing (ECFF) mechanism to improve the YOLOv8m network model structure. That is, the information entropy distribution of the feature map is used as a physical constraint, and the high-entropy critical region, such as the smoke edge, is identified by constructing an Entropy Topology Map, forcing the feature mapping to obey the entropy constraint rule so that the network focuses on the information-critical state region; the model’s ability of sensing the phase change process of the smoke in the mine is improved.

**Figure 5:**
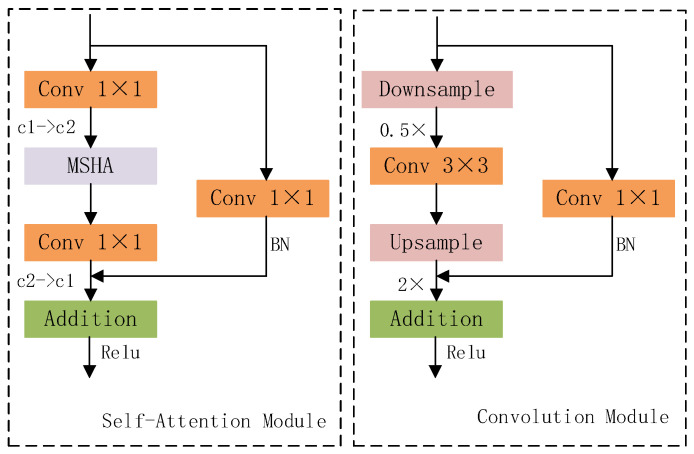
Self-attention and convolutional modular network structure.

Therefore, we utilize the HAT attention mechanism (super-resolution attention mechanism) to replace the v8 detector head to form a brand-new super-resolution detector head, the HATHead module. The HATHead module consists of self-attention and convolutional modules, the structures of which are shown in Figure 5 for two types of neural network modules.

Hybrid Attention Transformer (HAT) enhances the performance of single-image super-resolution reconstruction by fusing the channel attention and self-attention mechanisms, which effectively integrates the global pixel information and effectively improves target detection feature expression.

**Figure 6:**
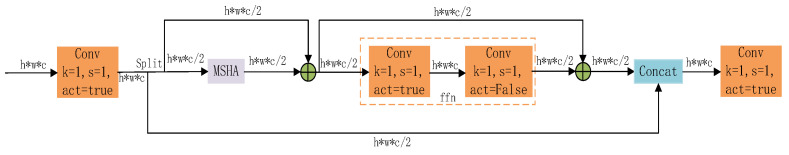
Structure of the self-attention mechanism (PSA).

We also modified the internal architecture of YOLOv8m by adding a self-attention mechanism to the backbone portion of the network (backbone) to give it superior inference capabilities. We propose the use of a single self-attention (PSA) module to improve the performance of the model without significantly increasing the computational cost. The network structure of the self-attention module is shown in Figure 6.

**Figure 7:**
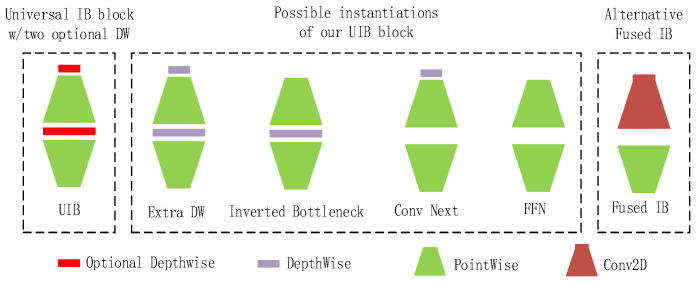
Universal Inverted Bottleneck (UIB) network architecture diagram.

Finally, we innovatively improved the C2f module in YOLOv8m by using the Universal Inverted Bottleneck (UIB) module from MobileNetV4 [38] to form the C2f_UIB module, which aims to model lightweight and high performance. Among them, the UIB network structure is shown in Figure 7.

### 4.2. Improved YOLOv8m Network Modeling Structure

**Figure 8:**
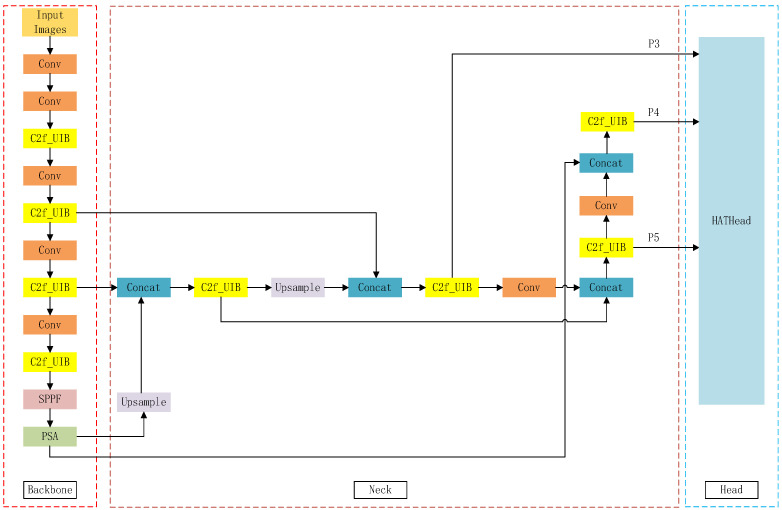
Structure of the YOLOv8m-HPC network model.

The above improvement strategy, as a whole, ensures that the model’s ability to detect both small and large smoke in images while maintaining low time complexity. The structure of the improved YOLOv8m-HPC network model is shown in Figure 8.

## 5. Construction of the MFSIDD Dataset

There are no natural light sources, such as daylight, moonlight, and starlight, in underground coal mines, and the overall brightness of the background environment is not as good as that on the ground due to underground lighting. The datasets used in the existing mine fire and smoke recognition algorithms are directly based on ground fire and smoke for modeling and testing, and so they do not fully consider the real underground background environment. The ground smoke dataset is usually based on natural lighting or standard lighting conditions, which are different from the lighting conditions of the underground environment; secondly, the background environment of the ground smoke dataset (e.g., sky, buildings, vegetation, etc.) is completely different from that of the underground environment (e.g., roadway, equipment, coal wall, etc.).

**Figure 9:**
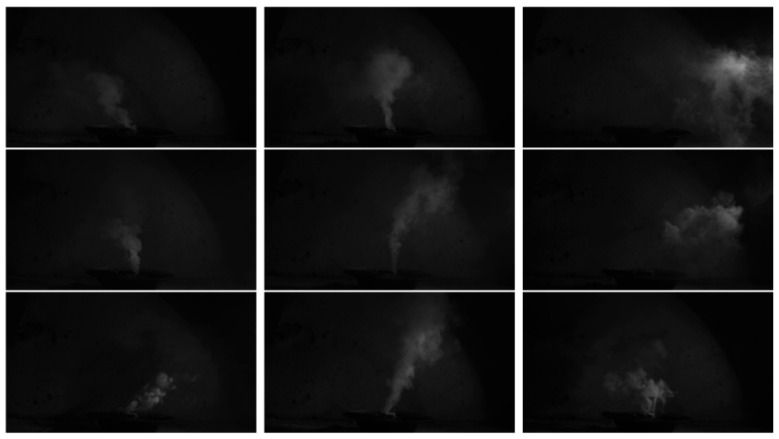
Smoke target image.

Therefore, it is necessary to consider the real underground background environment for the research of mine fire smoke detection and identification, and it is necessary to collect real or approximate smoke data samples of the underground environment for testing the method’s performance. We simulated the real underground background environment without daylight, moonlight, or starlight, and performed smoke image video acquisition to construct the Mine Fire Smoke Image Detection Dataset (MFSIDD), which contains 3119 smoke images; some of the smoke dataset images are shown in Figure 9.

**Figure 10:**
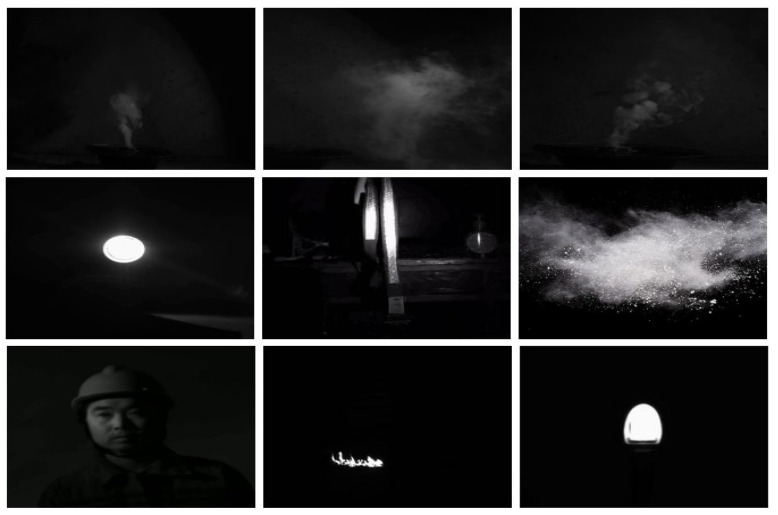
Partial image of the MFSIDD dataset.

The 3119 smoke images were labeled using labelimg (official version v1.8.1) software. Secondly, in order to ensure the anti-interference ability of the algorithm’s recognition, the authors added another 690 interference images, such as pedestrians, artificial light sources, reflective objects (belts, etc.), dust, and other interference images, to the dataset. The total number of labeled dataset images is 3809. The ratio of smoke to its interfering images is about 4.5:1. The dataset is randomly categorized according to 6:2:2 and is used as the training and testing of the smoke recognition model of this paper’s method in order to validate the reasonableness of the proposed method in this paper. Some images of the MFSIDD dataset are shown in Figure 10.

## 6. Test Results and Analysis

### 6.1. Algorithm Performance Evaluation Metrics

In order to intuitively and objectively evaluate the detection performance of the proposed algorithm for mine fire smoke images, this paper analyzes and compares the detection performance of mine fire smoke images using different index values.

In this paper, three evaluation metrics: precision–recall (*P*–*R*) curve, average precision (*AP*), and mean average precision (*mAP*) were selected for the MFSIDD dataset with the following formulas:

(8) $$P=\frac{T_{p}}{T_{p}+F_{p}}\times 100\%,$$

where *P* is the smoke image recognition accuracy; *T_p_* is the number of correctly recognized images as smoke images; and *F_p_* is the number of incorrectly recognized images as smoke images.

(9) $$R=\frac{T_{p}}{T_{p}+F_{N}}\times 100\%,$$

where *R* is the smoke image recognition recall rate; and *F_N_* is the number of unrecognized smoke images.

(10) $$AP=\int_{0}^{1}P(R)dR,$$

where *AP* is the average accuracy of smoke image recognition; and *P* (*R*) is the expression of the function, with recall rate R as the independent variable.

(11) $$mAP=\frac{1}{N}\sum_{i=1}^{N}AP_{i}$$

where *AP_i_* is the average accuracy of recognition for the ith category in the MFSIDD dataset; and *N* is the number of target categories in the data sample.

### 6.2. Analysis of Target Detection Results

**Figure 11:**
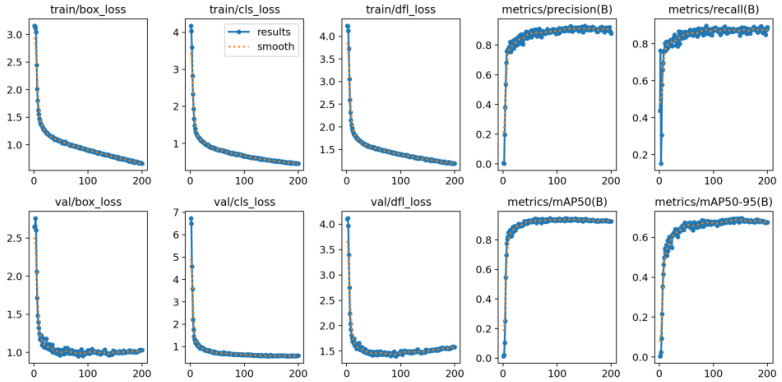
YOLOv8m-HPC model training and validation results.

Under the WINDOWS environment, the hardware configuration includes an Intel (R) Core (TM) i7-10750H CPU processor, 16 GB RAM, and a Nvidia GeForce RTX 1650 graphics card. The software configuration uses Python 3.8 and pytorch 1.9.0 to build the improved YOLOv8m-HPC algorithm model, and the classified MFISDD dataset is used to train the model, with a set iteration number of 200, a momentum factor of 0.937, an initial learning rate of 0.01, and a training batch of 4. The results of the model training and validation are shown in Figure 11.

**Figure 12:**
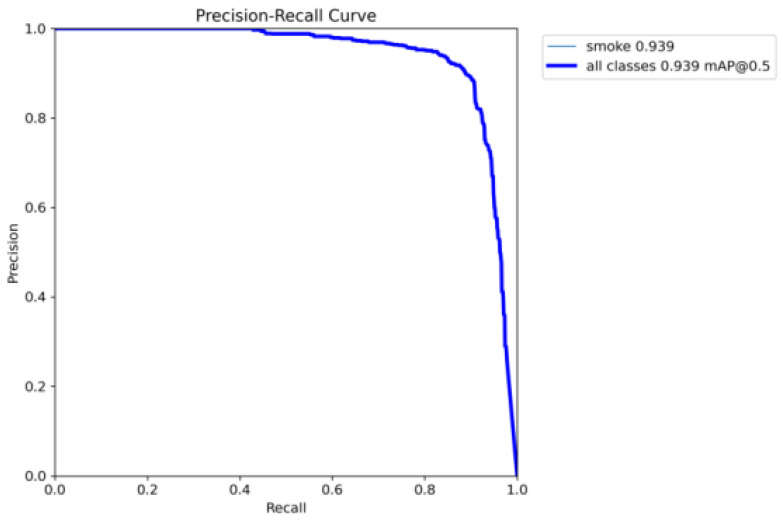
*P–R* curves of the YOLOv8m-HPC model on the test set.

The P–R curve of the YOLOv8m-HPC model on the test set is shown in Figure 12. Since the *mAP* value for mine fire smoke image recognition is obtained by calculating the area enclosed by the P–R curve and the axes, the larger the area, the better the *mAP* value and the higher the recognition accuracy. Combined with the *P–R* curve in Figure 12, it can be seen that the *mAP* value for smoke recognition is 0.939, and the model recognition effect is better.

**Figure 13:**
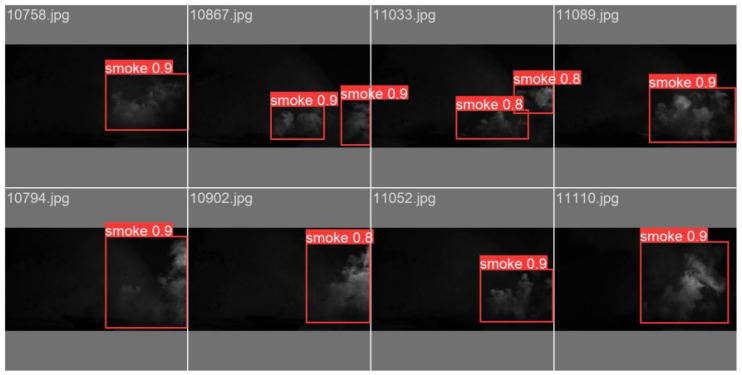
Smoke target image recognition results.

The recognition results of the smoke target image are shown in Figure 13; the recognition is more accurate. This shows that the YOLOv8m-HPC image recognition algorithm proposed in this paper performs well when dealing with smoke targets.

### 6.3. Comparative Analysis of Ablation Experiments and Algorithms

**Figure 14:**
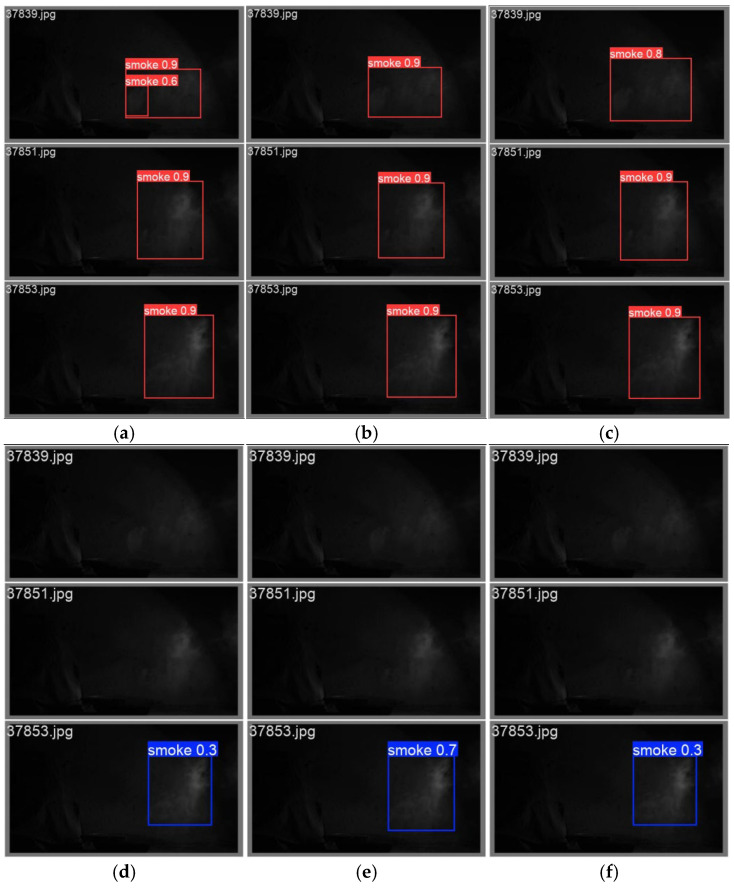
Comparison of smoke target image recognition algorithms. (**a**) Our methodology detection; (**b**) YOLOv8m detection; (**c**) YOLOv5m detection; (**d**) YOLOv9m detection; (**e**) YOLOv10n detection; (**f**) YOLOv11n detection.

As shown in Figure 14, the results of smoke target image recognition of three different methods (the present method YOLOv8m-HPC, the baseline model YOLOv8m, and the similar algorithm YOLOv5m) are demonstrated. Through comparative analysis, the following conclusions can be drawn: the present method (Figure 14a) has the highest recognition accuracy; it is able to monitor both large and small smoke targets and exhibits good overall performance. The baseline model (Figure 14b) and similar algorithms (Figure 14c) perform better in large smoke target recognition but worse in small smoke target recognition. From the comparative analysis of image recognition, it can be seen that this method can capture more detailed features of smoke, can monitor both large and small smoke targets, and shows better comprehensive performance than the baseline model and similar algorithms.

In order to further validate the performance enhancement of the improved YOLOv8m-HPC model, ablation experiments were carried out on the MFISDD dataset by setting the same parameter values for the improved YOLOv8m-HPC. Furthermore, a performance comparison with the existing SOTA algorithms, YOLOv5m, YOLOv8m, YOLOv9m, YOLOv10n, and the newest version, YOLOv11, in the YOLO family was undertaken to compare performances. The results are shown in Table 2.

As can be seen from the comparison of the results of the ablation experiments in Table 2, on the basis of the baseline model (Algorithm 2 in Algorithm 6), by adding a part of the attention mechanism to the backbone network, the recall rate of target detection, *R*, and the average detection precision, *mAP* (50–95), improved; in Algorithm 7, by adding an attention mechanism to the backbone network and improving the C2f module, the average detection precision, *mAP* (50), was further improved; in Algorithm 8, by adding the attention mechanism to the backbone network and the detection head, respectively, the target detection recall rate and the average detection precision *mAP* (50) improved. By fusing Algorithms 6, 7, and 8, this method, i.e., Algorithm 9, improves the average detection precision *mAP* (50) and *mAP* (50–95) even further, and the algorithmic model is optimal in its entirety. Therefore, the improved YOLOv8m-HPC model in this paper improves the recall and average precision of smoke recognition and increases the single-frame detection speed, where the Fps can be up to 25 frames per second, which can satisfy the demand of real-time detection and faster inference.

As can be seen from the comparison of the experimental results of similar algorithms in Table 2, compared with the baseline algorithm YOLOv8m, the improved algorithm reduces the precision rate by 0.8%, but we improved the recall rate, average detection precision *mAP* (50), *mAP* (50–95), and FPS by 2.3%, 0.4%, 1.6%, and 47%, respectively, through the optimization of the network model, which meets real-time detection demand. We believe that it is more critical to consider localization accuracy, recall (reflected in *mAP*), and real-time performance (FPS) together. This slight decrease in precision within an acceptable range is exchanged for a significant gain in *mAP* and speed, bringing substantial optimization to the overall performance of the algorithm. When compared to the similar algorithm YOLOv5m, the performance metrics of precision, recall, average detection accuracy (*mAP*) (50), and *mAP* (50–95) are improved by 5%, 3.1%, 2.9%, and 6.1%, respectively. Compared with the similar YOLOv9m algorithm, although there is a decrease of 8.0% in inference speed, there is an improvement of 4.6%, 9.6%, 5.0%, and 9.2% in the precision, recall, average detection accuracy *mAP* (50), and *mAP* (50–95) performance metrics, respectively. Compared with the similar YOLOv10n algorithm, it improves the precision, recall, average detection accuracy *mAP* (50), and *mAP* (50–95) performance metrics, respectively, by 13.2%, 13.8%, 10.7% and 12.0%, although it reduces the inference speed (12%). Compared to the similar YOLOv11n algorithm, although there is a 24% reduction in inference speed, it improved precision, recall, average detection accuracy *mAP* (50), and *mAP* (50–95) performance metrics, respectively, by 11.7%, 16.9%, 13.5%, and 18.5%.

Mine fire detection is a typical time-sensitive task, although the faster the inference is, the higher the precision, recall, and average detection accuracy *mAP*, which predicts that the better the model, the lower the false alarm rate and leakage rate of mine fire smoke detection. Therefore, in summary, the algorithm proposed in this paper is comprehensively optimal.

## 7. Conclusions

(1) According to the entropy change characteristics of spatio-temporal information brought by smoke diffusion movement and the entropy-invariant (or less change) characteristics of background interference information, based on the separation of spatio-temporal entropy, the method of entropy enhancement of isometric frame image differential fusion of smoke target images is proposed; this method effectively suppresses background noise and, at the same time, enhances the detail clarity of the smoke target image.
(2) The YOLOv8m-HPC model method for recognizing smoke target images is proposed. The target detection layer detection head was added to the attention mechanism and replaced with the super-resolution detection head HATHead module, which effectively improves the target detection feature expression capability. Using the self-attention mechanism (PSA) module, the performance of the model is improved without significantly increasing the computational cost by improving the C2f module to the C2f_UIB module. This strategy ensures the improved detection ability of the proposed model in recognizing small and large smoke in images.
(3) The experimental results show that the improved YOLOv8m-HPC model in this paper improves the recall and average precision of smoke recognition and increases the single-frame detection speed. The fps can reach 25 frames, which can satisfy the demand of real-time detection and faster inference. Compared to the YOLOv5m algorithm, it improves accuracy by 5%, recall by 3.1%, average detection precision *mAP* (50) by 2.9%, and *mAP* (50–95) by 6.1%. Compared to YOLOv8m, the algorithm improves 2.3%, 0.4%, 1.6%, and 47% on recall, average detection accuracy *mAP* (50), *mAP* (50–95), and FPS, respectively, although it decreases 0.8% on precision. Compared to YOLOv11n, although there is a 24% reduction in inference speed, improvements of 11.7%, 16.9%, 13.5%, and 18.5% are observed in the precision, recall, average detection accuracy *(mAP)* (50), and *mAP* (50–95) performance metrics, respectively. The YOLOv8m-HPC recognition method proposed in this paper has the best performance compared to similar algorithms and baseline algorithms.

## Figures and Tables

**Table 1:** Subjective user study MOS results.

Method Category	Background Subtraction Method	Frame Difference Method	Our Method
Expert 1	9	1	14
Expert 2	8	2	15
Expert 3	6	1	14
Expert 4	8	1	13
Expert 5	7	2	14
Expert 6	11	3	15
Expert 7	7	1	15
Expert 8	7	1	14
Expert 9	9	1	15
Expert 10	6	2	14
Expert 11	6	1	13
Expert 12	8	1	12
Expert 13	10	1	11
Expert 14	10	2	15
Expert 15	8	1	14
Total score of judging (*T_gs_*)	120	21	208
The mean opinion score (*MOS*)	40.0	7.0	69.3

**Table 2:** Comparison of ablation experiments and algorithms.

Serial Number	Algorithm Type	*P*/%	*R*/%	*mAP* (50)/%	*mAP* (50–95)/%	Fps/(f·s^−1^)
1	YOLOv5m	86.4	85.7	92.1	66.3	16
2	YOLOv8m	92.2	86.5	94.6	70.8	17
3	YOLOv9m	86.8	79.2	90.0	63.2	27
4	YOLOv10n	78.2	75.0	84.3	60.1	28
5	YOLOv11n	79.7	71.9	81.5	53.9	31
6	YOLOv8m-PSA	91.9	86.1	94.3	72.4	18
7	YOLOv8m-PSA-C2f_UIB	91.8	89.9	94.9	71.8	19
8	YOLOv8m-PSA-HATHead	87.0	90.0	94.4	71.7	17
9	YOLOv8m-PSA-C2f_UIB-HATHead	91.4	88.8	95.0	72.4	25

## Data Availability

The original contributions presented in this study are included in the article. Further inquiries can be directed to the corresponding author.
